# Mapping the expression of an *ANK3* isoform associated with bipolar disorder in the human brain

**DOI:** 10.1038/s41398-022-01784-6

**Published:** 2022-01-28

**Authors:** Asbjørn Holmgren, Lars Hansson, Kristine Bjerkaas-Kjeldal, Agata Antonia Rita Impellizzeri, Gregor D. Gilfillan, Srdjan Djurovic, Timothy Hughes

**Affiliations:** 1 Department of Medical Genetics, Oslo University Hospital, Oslo, Norway; 2 Department of Medical Genetics, University of Oslo, Oslo, Norway; 3 NORMENT, Institute of Clinical Medicine, University of Oslo, Oslo, Norway; 4 NORMENT, Department of Clinical Science, University of Bergen, Bergen, Norway

**Keywords:** Molecular neuroscience, Bipolar disorder, Genetics

## Abstract

The gene *ankyrin-3 (ANK3)* has been consistently associated with bipolar disorder (BD) in several genome-wide association studies (GWAS). The exact molecular mechanisms underlying this genetic association remain unknown. The discovery of a loss-of-function variant (rs41283526*G) in an alternatively spliced exon (ENSE00001786716) with a protective effect, suggested that elevated expression of this particular isoform could be a risk factor for developing the disorder. We developed a novel approach for measuring the expression level of all splice forms at a challenging genetic locus using a combination of droplet digital PCR and high-throughput sequencing of indexed PCR amplicons. The combined method was performed on a large collection of 568 postmortem brain samples of BD and SCZ cases and controls. We also studied the expression of the splice forms in a child-development cohort of 41 healthy males. We found that our approach can quantify the splice forms in brain samples, although with less precision than ddPCR. We detected highly significant differences in expression of splice forms and transcription start sites between brain regions, notably with higher expression of the BD-associated isoform in the corpus callosum compared to frontal tissue (mean fold change = 1.80, *p* < 1e-4). Although the patients in our sample expressed the BD-associated splice form at a similar level to controls, adolescents in our child-development cohort had a clearly higher expression level than younger children (mean fold change = 1.97, *p* = 5e-3). These results suggest that this *ANK3* splice form may play a role in the myelin maturation of the human brain.

## Introduction

Bipolar disorder (BD) is a psychiatric illness with a prevalence of about 1% [1] characterised by severe mood swings between mania/hypomania and depression. BD has a heritability of 60–80% [2, 3], but its aetiology is highly polygenic [4, 5]. The latest genome-wide association study (GWAS) of this disorder, which combined 41,917 cases with 371,549 controls, found 64 genome-wide significant loci. However, individual loci have only small effect sizes: the highest odds ratio (OR) in the study was 1.15 for a SNP in the MHC-locus [6]. In combination, these loci are estimated to account for 18% of heritability (*h*^2^_SNP_) [6].

**Fig. 1:**
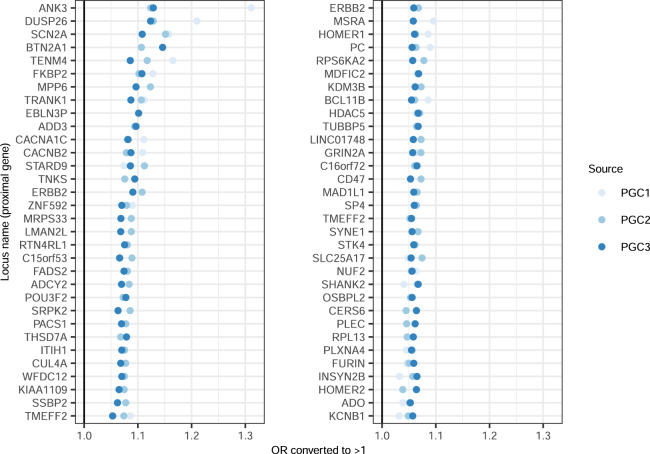
Odds ratios from all PGC GWAS of bipolar disorder. Odds ratio (OR) of the 64 loci with genome-wide significance in PGC3. OR values have been converted such that there is consistent use of reference and alternate alleles and all OR are greater than 1. Loci sorted by the median OR value across studies. Loci named with previously reported gene names (for published loci) or with nearest gene (otherwise).

There have been a total of three BD GWASs from the Psychiatric Genomics Consortium (PGC) [6–8], and in each case a locus containing the *ANK3* gene was genome-wide significant and had one of the largest ORs (Fig. 1). The encoded sub-membrane adapter protein ankyrin-G, is important for correct assembly of voltage-gated Na^+^ channels in axon initial segments and nodes of Ranvier in nerve cells [9, 10]. Ankyrin-G is also required for normal paranodal junction assembly in myelinating oligodendrocytes of the central nervous system [11]. However, the genetic association in itself does not shed much light on the exact molecular function that could elevate the risk of disease.

**Fig. 2:**
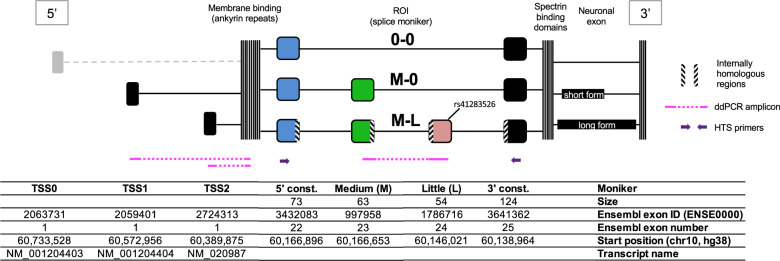
Exonic structure of the ANK3 gene and primer locations. Gene structure highlighting the TSS and genomic ROI and based on the transcripts in the RefSeq database. The protective SNP, rs41283526, is also indicated. Black TSS active in brain tissue, grey TSS inactive. A fourth isoform (0-L) was detected at very low levels but is not shown here. Figure not to scale.

In previous work, we identified a loss-of-function splice site variant (rs41283526*G) located in an alternatively spliced exon (ENSE00001786716) of a minor isoform of *ANK3* (Fig. 2*)*, that had a protective effect against BD and schizophrenia (SCZ) [12, 13]. Further, brain development data from Brainspan, suggested that this particular exon was not expressed in childhood, but began transcription in adolescence, which is when many patients enter the prodromal phase [14]. These findings suggest that elevated expression of this particular transcript could be a specific molecular risk factor for the disease. The finding that BD type 1 and SCZ patients had higher expression of this transcript in blood strengthened this hypothesis [15].

The exon bearing the loss-of-function variant is located in a wider alternative splicing context for which we introduce a specific nomenclature (Fig. 2). In this genetic region of interest (ROI), we call the two alternatively spliced exons ENSE0000997958 “M” (medium) and ENSE00001786716 “L” (little), attributing to their relative size. We name the specific splice forms using the exon name abbreviations. For example, a splice form containing both exons is coined “M-L” whereas one containing only M is coined “M-0”. There are three recognised transcription start sites (TSS) for *ANK3*, two of which have been shown to be active in the brain [15], and herein called TSS1 and TSS2.

Here, we extend our previous work in several ways. First, we studied the expression of the *ANK3* transcripts in the disease-relevant tissue by obtaining postmortem brain samples from patients and controls. We chose the frontal cortex (FC) and orbitofrontal cortex (OFC) regions because it is in this region that data from the Brainspan database indicate the clearest “switching-on” of the exon of interest during adolescence [15]. We also selected the corpus callosum because it is in this brain region that the exon of interest is most strongly expressed [12, 16]. By incorporating several hundred samples in our study, we aimed to have the statistical power to detect even small differences in expression. Since we are interested in the relative expression of several isoforms which differ at non-adjacent locations, we would ideally have measured the expression of specific full-length RNA transcripts, but this is not feasible to do at scale for long transcripts with current technologies. We, therefore, chose to measure the TSS and ROI separately. Second, in order to maximise the detection of rare transcripts, we used droplet digital PCR (ddPCR) to measure splice forms of *ANK3* in the ROI and TSS (Fig. 2). However, since not all splice forms of interest can be measured by ddPCR (because of the presence of two homology regions), we devised a method for quantifying the splice forms by amplifying the ROI with PCR and sequencing the amplicons with high-throughput sequencing (HTS).

The overarching aim of the study is to advance our understanding of *ANK3* transcript expression in the human brain, both in a case-control cohort and in a developmental cohort, providing insight into the molecular basis of the association between *ANK3* and BD.

## Materials and methods

### GWAS summary statistics

Summary statistics from the three bipolar disorder GWASs performed by the Psychiatric Genomics Consortium were downloaded from their website [17]. We began with the list of lead SNPs for the 64 genome-wide significant loci identified in PGC3, which is the latest and most highly powered study. Then, we extracted the OR and tested allele of these SNPs from each of the three studies. OR values were converted such that there was the consistent use of reference and alternate alleles across studies and all odds ratios were greater than 1, and then sorted by median OR across studies (Fig. 1, scripts available on github.com/asbhol).

### Samples

For the case-control samples, total RNA from corpus callosum, orbital-frontal cortex and frontal cortex was obtained from the Stanley Medical Research Institute: Array collection (105 individuals) and New collection (57 individuals). We also obtained 206 frozen tissue blocks of corpus callosum from the Harvard Brain Tissue Resource Center and the University of Pittsburgh Brain Tissue Donation program, and extracted RNA in-house. For the age-series samples, 41 frozen blocks of orbital-frontal cortex from healthy males aged 1–24 were obtained from the Maryland Psychiatric Research Center (MPRC) collection and extracted in-house (Table 1).

**Table 1:** Overview of brain samples (collections and brain regions).

	Child development	Case-control			
Brain region	Orbital-frontal cortex^a^	Orbital-frontal cortex^b^	Frontal cortex^b,c^	Corpus callosum^b^	Corpus callosum^d,e^
Sample type	Frozen tissue block	RNA	RNA	RNA	Frozen tissue block
Healthy controls	41	34	51	35	88
Bipolar disorder	0	32	53	34	45
Schizophrenia	0	34	54	35	73
Mean age	1–2 individuals from each age 1–24 years	44.1	45.6	44	61.1
Male prop	1	0.64	0.65	0.65	0.60
Female prop	0	0.36	0.35	0.35	0.40

Brain repository.

^a^Brain and tissue bank—University of Maryland.

^b^The Stanley Medical Research Institute, Array collection.

^c^The Stanley Medical Research Institute, New collection.

^d^Harvard Brain Tissue Resource Center.

^e^The University of Pittsburgh Brain Tissue Donation program.

### RNA extraction

RNA had already been extracted from the samples from the Stanley Medical Research Institute. We performed RNA extraction on the Maryland, Harvard and Pittsburgh samples with the TRIzol method according to the manufacturer’s protocol (Invitrogen, Thermo Fisher Scientific, Waltham, MA, USA). Briefly, 30–100 mg frozen tissue was added to 1 mL ice-cold TRIzol reagent (Invitrogen) and homogenised with a TissueRuptor II (Qiagen, Hilden, Germany). After incubation for 5 min, 0.2 mL chloroform (Sigma–Aldrich, Saint-Louis, MO, USA) was added, and the samples were incubated at room temperature for 2–3 min and centrifuged for 5 min at 12,000 × *g* at 4 °C. The aqueous phase was transferred to a new tube, and 0.5 mL isopropanol (Sigma–Aldrich) was added. The samples were incubated at room temperature for 10 min, followed by centrifugation at 12,000 × *g* for 15 min at 4 °C. The pellet was washed with 75% Ethanol (Sigma–Aldrich), air-dried and resuspended in 20–50 μL nuclease-free water (Qiagen). The RNA was further purified with RNeasy MinElute Cleanup Kit (Qiagen), and eluted in 20 μL nuclease-free water. The concentration of the RNA was measured with Quant-iT BR RNA Assay Kit (ThermoFisher Scientific), and integrity was assessed on an Agilent 2100 Bioanalyzer using RNA 6000 Nano chips (Agilent Technologies). Specimens with RNA integrity number (RIN) < 6 were omitted from the experiments. cDNA synthesis was performed on 200 ng of each sample with the High-Capacity cDNA Reverse Transcription Kit (Applied Biosystems, Thermo Fisher Scientific), using 20 μL reaction volume.

### Droplet digital polymerase chain reaction (ddPCR)

Droplet digital PCR (ddPCR) of the M-L splice form, containing both the “medium” and “little” exons, was performed with forward primer TGCTCAGTGATGGCGAATATATCT, reverse primer CCGTAAACTCTTGTACCTTGGGAAT, and FAM-labelled probe ATGTTGAAGAAGGTAATAGATG (Fig. 2), in a duplex with the endogenous control gene *GUSB* (TaqMan Assay ID Hs99999908_m1; primer limited assay with VIC-labelled probe). ddPCR for the transcriptional start sites (TSS) was performed with forward primer ATACCGGCGGTGATCTGTCT, reverse primer GCTCTTAAGTAACTTGCATTGGC, and FAM-labelled probe AAAACCTGCTCATAGGAA for the NM_001204404 isoform (TSS1); forward primer CAATGCTGAAGAAGAGCCTGAG, reverse primer AGGGCCTTTTCAAGGTGTCC, and VIC-labelled probe ATGCATTATGGCTCATGC for the NM_020987 isoform (TSS2). Assays were run in triplex with the endogenous control gene *GUSB* (TaqMan Assay ID Hs99999908_m1; FAM-labelled probe). The ddPCR reactions were prepared using 1× ddPCR Supermix for probes (no dUTP) (Bio-Rad), 0,9 µM of forward and reverse primer, 0,25 µM of probe, 1× assay mix of *GUSB* or *GUSB* primer limited, and 10 ng of cDNA. Droplets were generated on the QX200 AutoDG Droplet Generator (Bio-Rad). The plate containing the droplets was then transferred to a thermal cycler, and subjected to the following PCR profile suggested by the manufacturer: an initial melting step at 95 °C for 10 min; then 40 cycles of 94 °C for 30 sec, 60 °C for 60 sec, then 98 °C for 10 min and a 4 °C infinity hold. The plate containing the droplets was then placed in a QX200 Droplet Reader (Bio-Rad), which analyses each droplet individually using a two-color detection system able to detect the fluorescence from the FAM and VIC probes. After droplet reading, data analysis was performed using QX Manager Standard Edition v1.1 software (Bio-Rad). We did not measure TSS0, since we have previously shown that this TSS is not active in brain tissue [15].

### Amplification of ROI and HTS

cDNA-specific primers were designed to amplify the region of interest in the *ANK3* gene (Fig. 2). The forward primer, complementary to the constitutive exon ENSE00003432083, was the same in all *ANK3* PCR reactions. The reverse primer, complementary to the constitutive exon ENSE00003641362, included a 3’ tail with 16 different internal barcodes (8 nt) to allow for sample indexing. A similar oligo design was used for the endogenous *GUSB* gene: The forward primer was the same in all reactions, and the reverse primer included the same internal barcodes as for *ANK3*. All primers included a random quatromer at the 5’ end to increase diversity in the sequencing reads (Table S1). A Biomek FXP pipetting robot was used to set up the PCR reactions in 384-well plates, and the following program was used for amplification with Platinum SuperFi PCR Master Mix (Invitrogen): 98 °C for 30 s initial melting, 32 cycles of 98 °C for 10 s, 60 °C for 10 s and 72 °C for 15 s and finally 72 °C for 5 min. Products were verified on a 2100 Bioanalyzer (Agilent) with DNA 7500 chips. The full volume (10 μl) of each of the 16 reactions with different barcodes were then pooled and cleaned with 1.8× volume of Agencourt Ampure XP (Beckman Coulter). A fixed volume (5 or 10%) of the *GUSB* pooled PCR reaction was added to each corresponding samples’ *ANK3* pool before library preparation.

In order to quantify and adjust for bias against longer fragments (incorporating more or longer exons) in the PCR-amplification and in Illumina sequencing bridge-PCR, we ordered oligonucleotides of the 4 *ANK3* splice-form products and the *GUSB* endogenous control from Integrated DNA technologies (Coralville, IA, USA) as gBlocks Gene Fragments of length 125, 156, 210, 219 and 273 bp (Table S2) and quantified the template dilutions with ddPCR (details in supplementary materials). We then created six equimolar mixtures of the 5 oligos with a different template amount in each mixture (200 replicated twice, 800, 1600 and 3200 molecules). These mixtures were included in the PCR as separate samples and prepared for high-throughput sequencing along with the brain samples.

Pools of amplicons, with barcodes from 16 different individuals were normalised by concentration as measured by Quant-iT HS DNA Assay Kit (ThermoFisher Scientific) and prepared for sequencing with the Thruplex DNA-seq kit (Takara Bio, Shiga, JP). These pools contained both the full volume of *ANK3* amplicons and a lesser volume of the *GUSB* endogenous control amplicon. The prepared samples were sequenced on an Illumina Miseq platform (Illumina, Inc) with an average 1,244,798 reads per sample for the child-development samples (v2 micro flow cell, 300 bp single-end reads) and 471,063 per sample for the cases-controls (v3 flow cell, 300 bp paired-end reads). The quality of the sequenced data was verified with FastQC (v. 0.11.8).

Sequence data were processed using custom shell scripts. In brief, low-quality reads were trimmed and Illumina sequencing adapters were removed using Cutadapt (v. 3.3). The same software was used for demultiplexing based on the presence of internal barcodes (maximum error rate = 0.15), and paired reads merged using FLASH (v. 1.2.11). Cutadapt was used to identify which splice form was present in a read (maximum error rate = 0.10). The number of matches for each splice form was counted using a custom Awk script, and visualisation of results and statistical analysis was performed in RStudio (packages tidyverse, olsrr, ggstatsplot, rstatix and ggpubr). In order to account for yield differences between pooled samples after sample preparation for HTS, read numbers for each sample were normalised with average reads in the pool. All code and scripts are available on github.com/asbhol.

## Results

Droplet digital PCR (ddPCR) has been shown to be more precise and reproducible in measuring cDNA levels than that of the previous gold standard technique, quantitative PCR (qPCR), especially for rare transcripts [18]. Therefore, we describe the ddPCR results first. It is important to note that due to homology in the coding sequence flanking rs41283526*G (the loss-of-function variant with a protective odds ratio for BD), it was only possible to design a specific probe for the M-L splice form in the ROI, thus only this splice form of the ROI can be measured by ddPCR (in addition to the TSS1 and TSS2). It is because of this limitation that we designed the novel HTS approach to measure the levels of all splice forms in the ROI.

### Droplet digital PCR measurements

#### Expression differences between brain regions (case-control dataset)

**Fig. 3:**
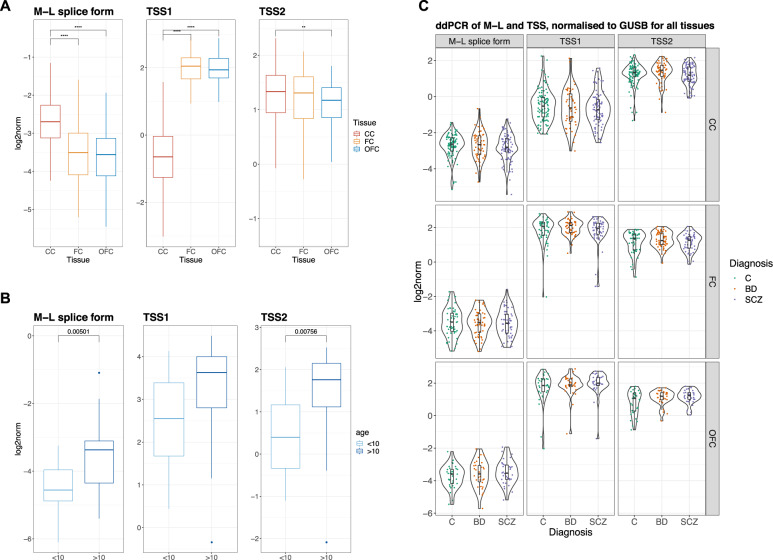
ddPCR measurements in brain regions and age groups. **A** Expression of the M-L splice form, TSS1 and TSS2 with ddPCR on the case-control sample set. Molecules per μL normalised to *GUSB* (log2-scale). Statistical testing with ANOVA and Tukey’s honestly significant difference (HSD) post-hoc test (**** *p* < 0.0001, ** *p* < 0.02). **B** Boxplot of ddPCR measurements of the M-L splice variant, TSS1 and TSS2 in the child-development set. Samples have been grouped into below and above 10 years old. Molecules per μL normalised to *GUSB* (log2-scale). *t*-tests of the difference in mean between age groups with unadjusted *p*-values for each target are indicated. **C** ddPCR measurements of the M-L splice variant, TSS1 and TSS2 in the case-control sample set, showing all tissues and diagnostic group. Molecules per μL normalised to *GUSB* (log2-scale).

We tested for differences in expression of the M-L splice form, TSS1 and TSS2 between brain regions by one-way ANOVA (Fig. 3A and C). The M-L splice form had higher expression in the corpus callosum than in the frontal cortex (FC) (*p* < 0.0001) and orbitofrontal cortex (OFC) (*p* < 0.0001). On the other hand, TSS1 had lower expression in CC than in FC (*p* < 0.0001) and OFC (*p* < 0.0001). TSS2 had higher expression in CC than OFC (*p* = 0.01). In the analysis of M-L expression, we noticed a larger variation in the BD subjects, but the variance was not different with statistical significance (Levene’s test, *p* = 0.117).

#### Changes in expression during child development and aging (both datasets)

The M-L splice form was measured in the OFC of healthy males aged 1–24 years old (*n* = 41) with droplet digital PCR (ddPCR). There was a significantly higher expression in individuals above 10 years old, compared to younger subjects, as assessed with a Student’s t-test on log-transformed measurements (group means −4.51, −3.53, p.adj 0.015). Similarly, the TSS2 had higher expression in the older age group (group means 0.517, 1.484, p.adj 0.028). TSS1 showed no significant difference between the age groups (p.adj 0.219). (Fig. 3B).

We also modelled the effect of age on M-L expression in the CC in the older case-control cohort (mean age: 55.4) by fitting a multiple linear regression model of expression as a function of diagnosis, RNA integrity number (RIN), postmortem interval (PMI), age and gender. The model indicated that M-L expression decreased with age in this elderly cohort (t = −3.744, *p* < 0.001) and increased with RIN (t = 2.434, *p* < 0.02), and these variables explained a significant proportion of the variance in expression of the M-L splice form. An identical model of TSS2 expression also showed a decrease with age (t = −2.569, *p* = 0.0108) and increase with RIN (t = 5.125, *p* = 6.15e-07).

#### Differences between cases and controls (case-control dataset)

We did not detect any statistically significant differences in the means of expression between any of the diagnosis subgroups (HC, BD, SCZ) with one-way ANOVA for the M-L splice form, TSS1 nor TSS2 as measured with ddPCR (Fig. 3C). Nor were there any statistically significant differences in the variance between diagnosis group for the M-L splice form, TSS1 nor TSS2 in any of the tissues, using Levene’s test for equality of variances.

### HTS measurement of splice forms

In order to measure splice forms that were not amenable to ddPCR measurement, we designed a method to sequence and quantify PCR amplicons in the ROI. ddPCR is considered a gold standard quantification method, thus for the M-L splice form, as well as the endogenous control *GUSB*, our measurements with both ddPCR and HTS of amplicons could be compared to evaluate the accuracy of our HTS method. We found that in the case-control dataset (*n* = 558), the Spearman correlation coefficient between the two methods was 0.71 for the M-L splice form and 0.43 for *GUSB* (both *p*-values < 2.2e-16) (Fig. S1).

The correlation for the M-L splice form was therefore satisfactory, but the *GUSB* correlation was weak. Thus, the *GUSB* measurement could not be used to normalise expression levels of the splice forms of interest. Without this normalisation, we were unfortunately unable to test for differences in levels of a particular splice form between groups of individuals.

**Fig. 4:**
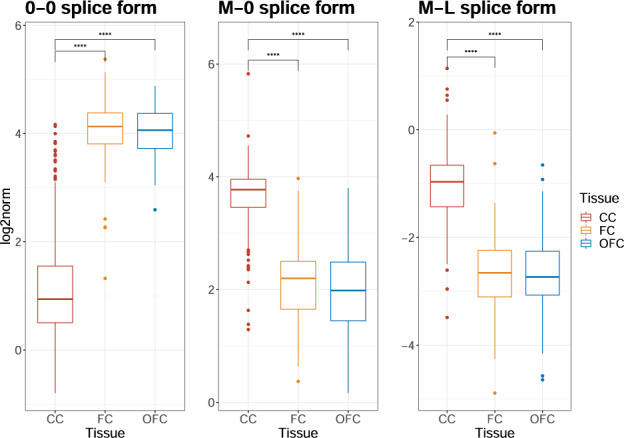
HTS measurement of different splice forms in ROI. Normalised expression of the splice forms 0-0, M-0 and M-L (Fig. 2) with HTS-sequenced amplicons on the case-control sample set (*n* = 568). Quantified reads normalised to *GUSB* (log2-scale). Amplicon length biases have been adjusted for (Fig. S2). Statistical testing with ANOVA and Tukey’s honestly significant difference (HSD) post-hoc test (**** *p* < 0.0001).

However, our HTS expression measures could be used to estimate the relative average abundancy of the different splice forms as they are not reliant on the *GUSB* normalisation. Our artificial samples of equimolar mixtures of synthetic oligos of *ANK3* splice forms and *GUSB* (after normalising for the total number of reads in the sequencing pools) show consistent ratios between the oligos across different template amounts and thus can be used to correct for the PCR bias between PCR products (Fig. S2). We found that the 0–0 splice form is most common in cortical tissue (FC, OFC), whereas the M-0 form is dominant in CC (Fig. 4). The M-L splice form shows low expression in all tissues, but is higher in CC than in cortical tissue (FC and OFC). The 0-L splice form was detected in all tissues, but at minute levels (Fig. S3).

## Discussion

Previous results have pointed towards the reduced expression of the specific M-L splice form as having a protective effect against bipolar disorder and schizophrenia. In the present study, we employed ddPCR to accurately measure transcription of the M-L splice form in human brain tissue, as well as the two active TSS of *ANK3*. We also developed a method to quantify the full spectrum of *ANK3* splice forms in the ROI, as we hypothesised that the ratio between expressed splice forms could be relevant for the normal development or pathophysiology of the disorders. The main objectives were to investigate the transcription of these splice forms during child development and to compare SCZ and BD cases to controls.

A number of studies have leveraged expression quantitative trait loci (eQTLs) in order to find links between genetic loci indicated in GWAS and gene expression [19]. There is growing evidence that such links are specific to certain tissues [20], and *ANK3* transcripts are expressed at different levels in different regions of the brain [21]. In order to investigate how the splice forms of the *ANK3* gene are involved in disease pathophysiology, we need to evaluate how these transcripts are expressed in brain regions with distinct cellular and neuronal circuit functions [13]. Therefore, we considered carefully which regions of the brain to investigate. Corpus callosum (CC) showed a relatively higher expression of the M-L splice form in published data [15] and this region of the brain is of particular interest since morphological abnormalities have been linked to both SCZ and BD [22, 23]. We decided to also include SCZ cases, since the expression of the M-L splice form in blood was significantly different in the blood of both SCZ and bipolar disorder 1 (BD1) patients [15]. Expression of the “little” exon (L) in the Brainspan database [24] was most pronounced in the orbitofrontal cortex (OFC), thus we chose this tissue for the child-development samples. Furthermore, lesions to this particular region of the brain can cause behavioural abnormalities that are somewhat similar to those observed in bipolar disorder patients, such as defects in analysis and integration of stimuli pertaining to real-life situations [25] and risk-seeking behaviour [26].

We could not measure all splice forms in the ROI by ddPCR because of stretches of sequence homology, so we attempted to develop a method for high-throughput sequencing of indexed amplicons. However, we observed a poor correlation between ddPCR and HTS measurement of our endogenous control *GUSB* (R = 0.43), thus removing the possibility of normalising our M-L measurement and testing for splice-form expression differences between diagnostic groups. We suspect that this poor correlation was due to the endogenous control in the HTS measurement having the added technical complexity of being run on a separate PCR reaction and added pool-wise in a lower volume to the total pool of amplicons to be sequenced. Fortunately, the correlation between ddPCR and HTS for the M-L splice form performed better in this respect (R = 0.71). We concluded that where the preferred method of ddPCR was not available, the HTS measures could be used to gauge average differences in expression levels between splice forms (after correction for PCR length bias using synthetic oligo samples), but with less precision and sensitivity than ddPCR.

The combination of the ddPCR and HTS methods provides a fairly clear picture of expression differences across brain regions. The M-L splice form of *ANK3*, clearly has a higher expression in CC than OFC, regardless of diagnosis. This is a confirmation of what has been seen before in control subjects when comparing transcription levels in different regions of the brain [15]. One possible explanation is that the CC is composed of white matter and contains a larger proportion of oligodendrocytes, further strengthening our interest in this cell type. Our splice-form quantification with HTS shows that the M-0 form also has a higher expression in CC, whereas the 0-0 form is most predominant in cortical tissue. A similar dichotomy is detected for the TSS with expression of TSS2 being higher in CC than FC (*p* < 0.05) and OFC (*p* < 0.01) whereas the TSS1 had lower expression in CC than in FC (*p* < 0.0001) and OFC (*p* < 0.0001). In combination, these results strongly suggest that M-0 and M-L are transcribed from TSS2 predominantly in white matter, whereas the 0-0 splice form is transcribed from TSS1 in cortical tissue. This may be driven by differences in cell composition in these brain regions, particularly neuron-oligodendrocyte cell count ratios.

Expression of the M-L splice form in adolescents and young adults (>10 years old) was clearly higher than in children in the child-development series. The same is true for TSS2, the transcription start site believed to be mainly expressed in oligodendrocytes. It is well established that the myelination process continues throughout adolescence and only completes in the early twenties [27]. It is possible that this development in expression is due to an increased proportion of oligodendrocytes in the brain samples. However, it is more likely due to individual cells increasing their expression of the M-L isoform, since the number of oligodendrocytes does not change appreciably beyond the first years of life [28]. Interestingly, in the much older case-control cohort, the multiple linear regression model on the CC samples indicated that in old-age, M-L expression decreases and age explains a statistically significant proportion of the variance in M-L expression. It is possible that changes in expression of disease-associated transcripts such as that observed here are part of the explanation for the decrease in symptom burden of bipolar disorder in middle- and old-age [29], but we do not have access to the detailed and longitudinal medical information needed to test this hypothesis in our study participants.

Given the previously reported differences of *ANK3* expression between cases and controls in whole blood [15, 30], and the fact that CC and FC brain tissue are arguably more relevant tissues than blood for psychiatric disorders, we hypothesised that we would observe statistically significant differences in the M-L splice form in the case-control cohort. However, tests in both the CC and FC failed to reject the null hypothesis (Fig. 3C, Table S3). It is entirely possible that the expression of these splice forms plays no role in the aetiology of the disorders. Alternatively, certain limitations must be reducing the power of our tests. For example, postmortem brains in case-control cohorts tend to be from older patients rather than from subjects in their youth when the disorder has its onset. Moreover, we observe a decrease in the expression of M-L in older individuals. Thus, it is possible that differences that were present in youth are no longer detectable. Another possible factor affecting power is that the technical variation in our measurements may swamp the biological variation. This could for example be caused by the need to normalise by an endogenous control such as *GUSB* which, although carefully selected for low variation in expression across individuals, is not free of such variation. If the true differences in expression levels between cases and controls are small, this variation may hide the true differences. Further, the fact that the transcripts we are measuring are very lowly expressed only exacerbates this problem. Such issues can only be overcome with better measurement technologies or greater sample sizes.

Other factors that could potentially be preventing us from detecting a true difference include the limited number of brain regions and disease heterogeneity. *ANK3* has different isoforms expressed in different cell types, and it could be the case that there is a dysregulation of *ANK3* isoform expression, but that it is occurring in a particular cell population or specific brain region other than those that we sampled in this study. Single-cell RNA sequencing (scRNAseq) and spatial transcriptomics could potentially overcome some of these limitations. However, the technology is still broadly restricted to measuring the aggregate gene expression of highly expressed genes; current protocols do not yield much information on the transcription levels of relatively lowly expressed or differentially spliced isoforms [31]. Psychiatric disorders can be heterogenous in their presentation, and there could be several subgroups experiencing different aetiologies. As an example, there was a difference on a group level between BD1- and BD2-diagnosed patients when it came to M-L expression in blood [15]. Thus, our analysis may have benefited from more detailed clinical information, such as the distinction between BD1 and BD2, but this was unfortunately not available.

In summary, we are able to detect highly significant differences in expression between brain regions, with higher expression of our BD-associated isoform M-L, as well as M-0, in the CC relative to FC/OFC. Similarly, at the TSS, TSS2 is more highly expressed in the CC relative to the FC/OFC, whereas TSS1 is less expressed. The CC consists primarily of myelinating oligodendrocytes, so our results suggest that these cells express the M-0 and M-L isoforms under the control of TSS2, whereas frontal tissue with its high neuronal cell composition has high expression of the 0-0 splice form from TSS1. Further, TSS2 and M-L both show an age-dependent expression form, which could be related to the maturating state of myelin during adolescence.

In the past, linkage studies and candidate gene investigations rarely led to significant and reproducible findings for genetic correlations with BD [32]. With the advent of large, well-powered GWAS, we now have consistent GWAS evidence of an association between *ANK3* and BD. Coupled with our own finding of an association between a functional SNP in a specific isoform of *ANK3* and BD, we investigated the expression of this isoform in search of a specific target. Such a target could significantly improve the likelihood of discovering a cellular phenotype for BD. However, despite the statistically strong genomic evidence, our measurement of specific splice forms, our use of state-of-the-art ddPCR gene expression measuring techniques in a large sample of human brain RNA, we did not detect any transcriptional differences between cases and controls. This suggests that it will be very challenging to follow up GWAS studies of bipolar disorder with RNA expression studies of single candidate genes or isoforms in the postmortem brain, and consideration of other methods or functional models is warranted [5, 33].

## Supplementary information

Supplemental Material
