# Supplementary material for: Mapping the expression of an ANK3 isoform associated with bipolar disorder in the human brain
Source: Transl Psychiatry. 2022 Jan 28;12:45. doi: 10.1038/s41398-022-01784-6 (PMC8799726; doi:10.1038/s41398-022-01784-6)
Supplement: Supplementary file 1 — Supplemental Material [file 41398_2022_1784_MOESM1_ESM.docx]

Supplementary

**Table S1 All primers used in this study**

**Table S2 Synthetic DNA oligos used as standards in the HTS quantification of oligos**

B

A


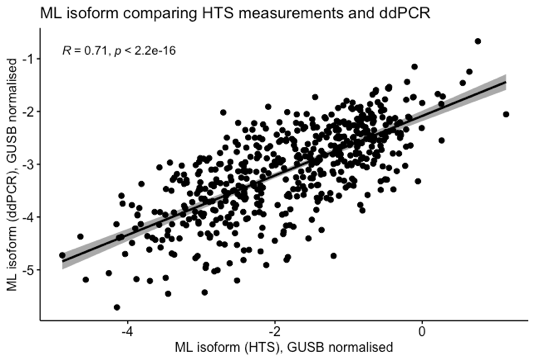


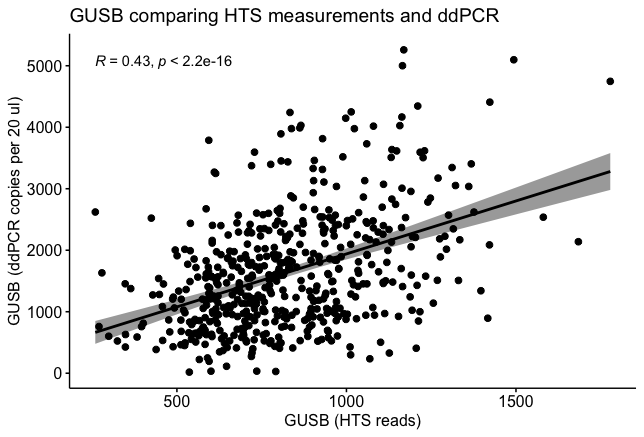


**Figure S1**

Scatter plot comparing the measurements of the M-L splice form (A) and the endogenous gene GUSB (B) with HTS and ddPCR in the case-control sampleset. Linear regression line (95 % confidence interval) and Pearson correlation coefficient is indicated.

A


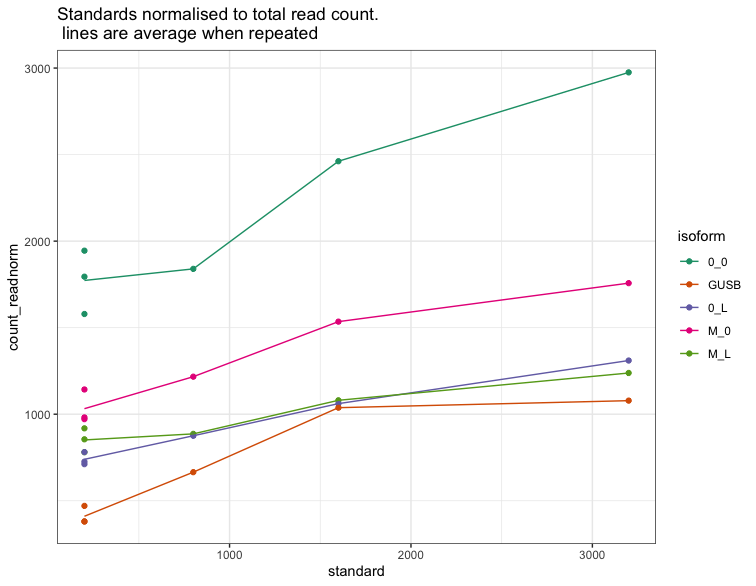


| splice form  B | amplicon size | size_ratio_ML | count_factor_ML |
| --- | --- | --- | --- |
| 0_0 | 172 | 0.60 | 0.46 |
| 0_L | 226 | 0.78 | 1.06 |
| M_0 | 235 | 0.81 | 0.76 |
| M_L | 289 | 1 | 1 |

**Figure S2**

A. HTS read counts of synthetic oligos mixed with several different, but internally equal, amounts of starting template. B. Ratios of size and read count of the different *ANK3* splice form synthetic oligos.

**Figure S3 HTS measurements of the 4 splice variants in the ROI in the case-control sample set.** Read counts have been normalised to total pool read count and GUSB reads on a log2-scale. Quantifications are adjusted for amplicon efficiency (figure S2)

**Table S3 Statistical testing (ANOVA) of ddPCR-quantified differences**.

Differences in expression of the TSS1, TSS2 and the splice form M-L between cases (BD/SCZ) and controls in all 3 tissue types. p-values have been adjusted for multiple testing using the Bonferroni method.


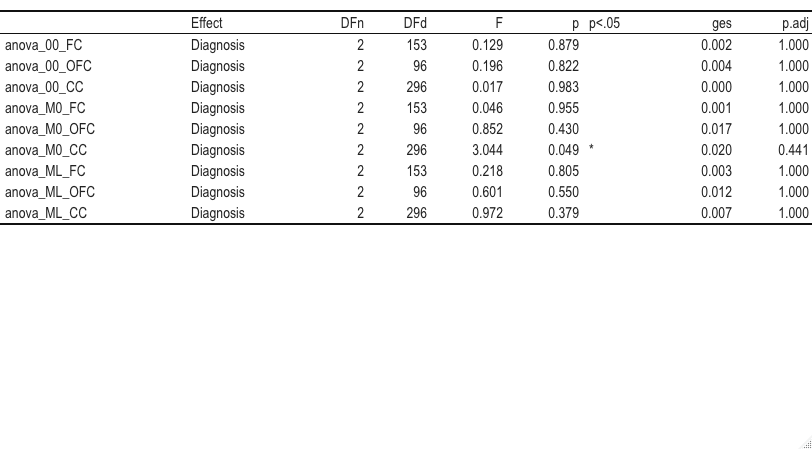


**Table S4 Statistical testing (ANOVA) of HTS-quantified differences**.

Differences in expression of the splice forms 0-0, M-0 and M-L between cases (BD/SCZ) and controls in all 3 tissue types. p-values have been adjusted for multiple testing using the Bonferroni method.

**Details on the accurate quantification of synthetic oligos before inclusion in the PCR for HTS amplification**

In detail, a 5 µl aliquot of the template dilutions were subjected to ddPCR. In one set of reactions, forward primer ACTGTCACAGAGAAGCACAA and reverse primer TGCAGGCAGGGAATCATCAC, targeting the constitutive exons flanking the ROI in *ANK3*, was combined with 1x EvaGreen master mix (Bio-Rad) to quantify the *ANK3* splice forms. In another set of reactions, forward primer CCACCTAGAATCTGCTGGCTA and reverse primer GTTGCTCACAAAGGTCACAGG, targeting the *GUSB* endogenous control gene, was combined with 1x EvaGreen master mix (Bio-Rad) to quantify *GUSB*. Both sets of reactions were combined in one plate, and droplets were generated on the QX200 manual droplet generator (Bio-Rad). It was then subjected to the following thermal profile: initial melting at 95°C for 5 min, then 40 cycles of 95°C for 30 sec and 60°C for 1 min, followed by a 4°C hold for 5 min and a 90°C hold for 5 min. Finally, a 12°C infinity hold was used. Results were read on the QX200 Droplet Reader (Bio-Rad), and analysed with QuantaSoft software (v1.7.4) (Bio-Rad).
